# KSHV and cancer: understanding the oncogenic machinery for next-generation diagnostic tools and therapies

**DOI:** 10.1007/s00203-025-04706-4

**Published:** 2026-01-21

**Authors:** João Vitor Geisteira Oliveira da Silva, Eidy de Oliveira Santos

**Affiliations:** 1 Programa de Pós-Graduação em Biomedicina Translacional (BIOTRANS), Afya-Universidade do Grande Rio (Afya-Unigranrio), Afya, RJ Brasil; 2 Universidade do Estado do Rio de Janeiro (UERJ), Rio de Janeiro, RJ Brasil

**Keywords:** KSHV (Kaposi's Sarcoma-Associated herpesvirus), Oncogenic mechanisms, Cancer hallmarks, Viral oncoproteins, Next-Generation therapies, Diagnostic tools

## Abstract

Kaposi’s sarcoma-associated herpesvirus (KSHV), or human herpesvirus 8 (HHV-8), is an oncogenic virus responsible for Kaposi’s sarcoma (KS) and lymphoproliferative disorders like primary effusion lymphoma (PEL) and multicentric Castleman disease (MCD). This review explores KSHV’s oncogenic mechanisms, focusing on its ability to manipulate host cell signaling, evade immune detection, and promote tumorigenesis through latent and lytic viral proteins. Key oncoproteins, such as LANA, vCyc, vFLIP, and vGPCR, activate cancer hallmarks, as sustained proliferation, immune evasion, angiogenesis, and resistance to cell death, by modulating pathways such as PI3K/AKT/mTOR and NF-κB. While histopathology and LANA staining remain diagnostic standards, emerging technologies, including advanced imaging and new molecular biomarkers, assay improved early detection. Of KSHV current therapies face challenges, especially in immunocompromised patients, highlighting the need for targeted treatments addressing viral infection. Next-generation approaches, such as CRISPR-Cas9 and therapeutic aptamers, aim to inhibit viral replication, modulate oncogenic pathways, and enhance immune responses. Current diagnosis of KS still relies primarily on histopathology and LANA immunostaining, which remain the gold standard but present important limitations, particularly in early or atypical lesions and in distinguishing latent from lytic infection. Despite advances in conventional chemotherapy and antiretroviral therapy, KSHV-associated malignancies lack virus-specific targeted treatments, and clinical outcomes remain suboptimal, especially in immunocompromised patients. By integrating emerging diagnostic biomarkers, such as viral microRNAs, with next-generation therapeutic strategies—including gene editing and synthetic biology-based approaches—this review highlights opportunities for precision medicine to improve disease detection, therapeutic specificity, and patient outcomes. Collectively, we provide a comprehensive framework for understanding KSHV-driven oncogenesis while outlining critical directions for future diagnostic and therapeutic innovation.

## Introduction

Human herpesvirus 8 (HHV-8) belongs to the Herpesviridae family and Gammaherpesvirinae subfamily (Fauquet 2005). HHV-8, also known as Kaposi’s sarcoma–associated herpesvirus (KSHV), is the etiological agent of Kaposi’s sarcoma (KS) and other lymphoproliferative disorders (DLPs), most notably primary effusion lymphoma (PEL) and multicentric Castleman disease (MCD) (Quadrelli 2011). In addition to these malignancies, studies have revealed associations with other diseases, such as multiple myeloma (MM), intravascular lymphoma (Gwiti et al. 2020), cervical carcinogenesis (Dai et al.), Kikuchi disease (Hudnall et al. 2008). Its viral architecture consists of an icosahedral capsid, a tegument layer, and a lipid bilayer (Spear and Longnecker 2003). The genetic material of KSHV is similar to that of other herpesviruses, consisting of a linear double-stranded DNA genome with an approximate size ranging from 165 to 170 kb. This genome contains a single central coding region (LUR) of approximately 145 kb, which harbors all viral reading frames, totaling around 100 open reading frames (ORFs) (Renne et al. 1996). These regions encode about 90 viral proteins and non-coding RNAs (ncRNA) and are flanked by long terminal repeats (LTRs) rich in GC (Mel 2020). The LTRs have ~ 801 base pairs, of which 85% are compounds of G and C that contribute to the high stability of viral DNA (Russo et al. 1996). In addition, KSHV encodes a unique set of genes (K1–K15) that play key roles in viral persistence, immune evasion, and tumorigenesis (Bala 2012; Lee et al. 1998). The latent viral genome has a circular episomal conformation and becomes linear during the lytic replication cycle (Renne et al. 1996; Mel 2020). Large-scale phylogenomic analyses reveal substantial genetic plasticity of KSHV. Analysis of over 2,100 global ORF-K1 sequences demonstrates extensive subtype diversification (A–F), driven by high recombination rates, with at least eleven major recombination events across the K1 locus. These findings indicate that most contemporary KSHV subtypes emerged within the last ~ 300 years, supporting rapid and ongoing viral evolution (Wang et al. 2025). Moreover, the differential gene flow observed among KSHV subtypes—particularly the high level of genetic recombination and inter-subtype gene exchange in subtypes A and C, compared with the more isolated evolution of subtype B—underscores the dynamic population structure of the virus. These genomic differences may influence viral transmissibility, tissue tropism, regional incidence patterns, and potentially KS aggressiveness and disease progression.

Since its discovery by MoritzKaposi in 1872 KS has been described as a pigmented multifocal sarcoma of the skin (Kaposi 1982). In the 1990 s, early epidemiological observations led to the attribution of HIV transmission—and consequently the high incidence of KS—predominantly to male homosexual populations. This interpretation, common at the time, later contributed to the recognition that KS was associated with an additional, sexually transmitted etiological agent independent of HIV. In 1994, a study aimed to identify unknown DNA sequences in HIV positive patients revealed KSHV DNA in KS tissues. Subsequently, the complete sequence of the KSHV DNA was identified, as well as its classification as an oncogenic virus (Chang et al. 1995). Following the identification of KSHV as an oncogenic virus, the epidemiological burden of KS became more clearly defined. Thus, according to health organizations, including the American Society of Clinical Oncology (ASCO) and the American Cancer Society, KS is the most frequent cancer—approximately 100-fold more common (Lagos and Boshoff 2007)—in HIV-positive patients, constituting one of the AIDS-defining malignancies alongside non-Hodgkin lymphoma (NHL) and cervical cancer (CC) (Lagos and Boshoff 2007; https 2021). In addition, multiregional comparative studies based on 200,000 HIV positive patients with highly active antiretroviral therapy (HAART), revealed an incidence of KS development in 198.6/100,000 inhabitants per year from 57 countries in the continents of North America, Latin America, Europe, Asia-Pacific and Africa southern (The AIDS-defining Cancer Project Working Group for IeDEA and COHERE in EuroCoord 2017).

**Fig. 1:**
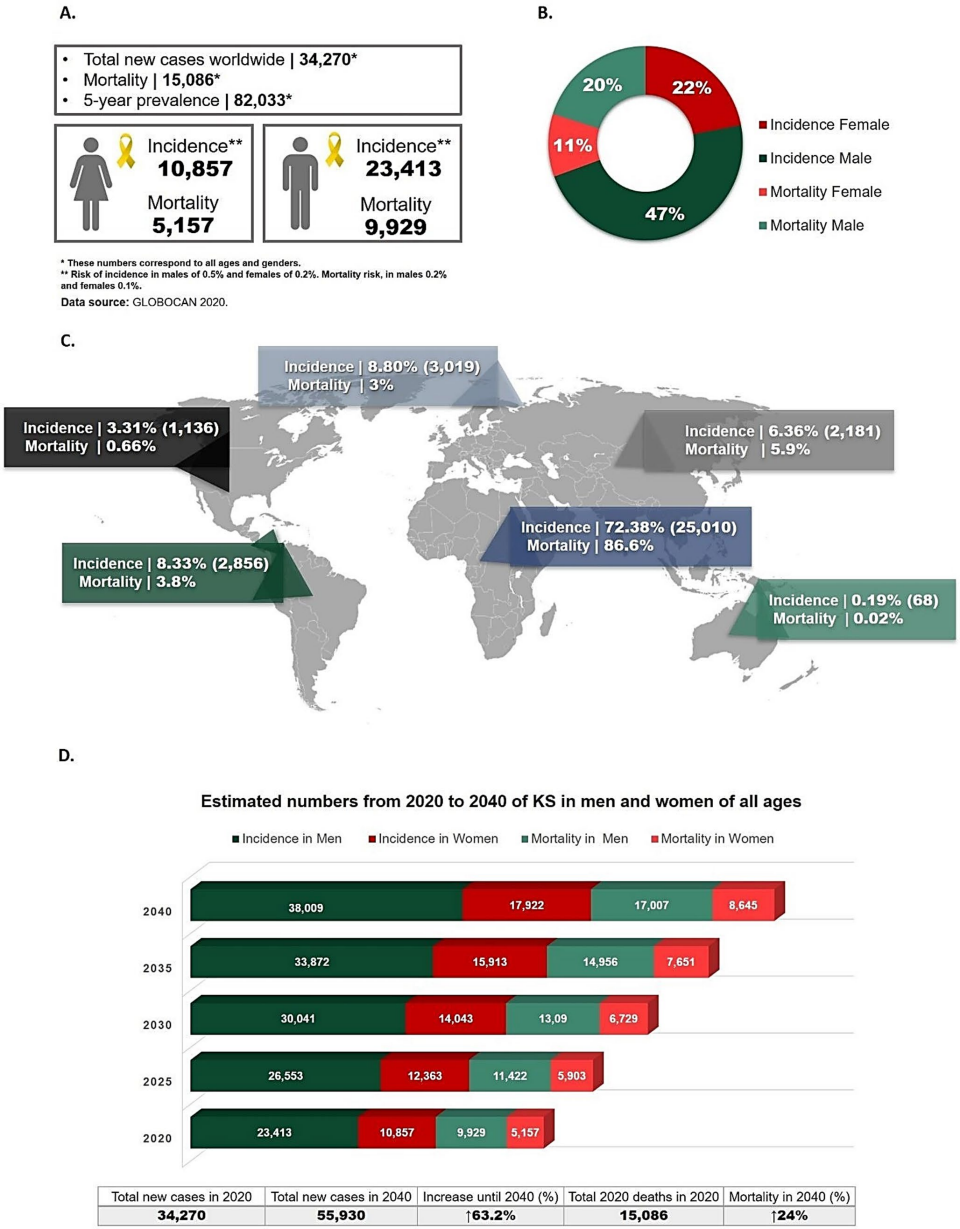
Statistical data on Kaposi’s sarcoma (**A**, **B**, **C**), and projections up to 2040 (**D**), by sex and age group. (**A**–**C**) Worldwide distribution of incidence and mortality by sex and geographic region based on GLOBOCAN 2020 data. (**D**) Projected incidence and mortality trends for KS from 2020 to 2040.All numbers and statistical data used to construct the graph were extracted from the updated database of the International Agency for Research on Cancer of the World Health Organization, GLOBOCAN 2020

According to the 2020 GLOBOCAN database, show new KS cases and deaths of 34,270 and 15,086, respectively, with mortality in ~ 44% of cases. The incidence and mortality rate is almost 2 times higher in men compared to women; and the worldwide geographic distribution data show that East, South and Central Africa are the endemic regions with the highest number of KS cases from both sexes (72.38% of incidence and 86.6% of mortality), with Uganda and Mozambique among the most affected countries (Sung et al. 2021) (Fig.1A–C). Projections of KS incidence and mortality from 2020 to 2040 estimate 55,930 new cases in 2040, corresponding to a 63.2% increase, while mortality is projected to rise by 24% (Fig. 1D). In Iran, a high seroprevalence of KSHV has been reported among patients undergoing hemodialysis (16.9%; *n* = 118), kidney transplant recipients (25%; *n* = 100), and HIV-positive individuals (45.7%; *n*= 35) (Jalilvand et al. 2011). In Brazil, children between 1 and 2 years old born to HIV positive mothers had a seroprevalence of 2%, while between 3 and 13 years old it was 8% (Machado et al. 2005).

KS remains one of the most prevalent malignancies affecting immunocompromised individuals, particularly people living with HIV. In this review, we discuss current and emerging biotechnological approaches for the diagnosis and treatment of KSHV-associated diseases and highlight how viral oncogenic factors activate key hallmarks of cancer.

## Kaposi’s sarcoma | KS

**Fig. 2:**
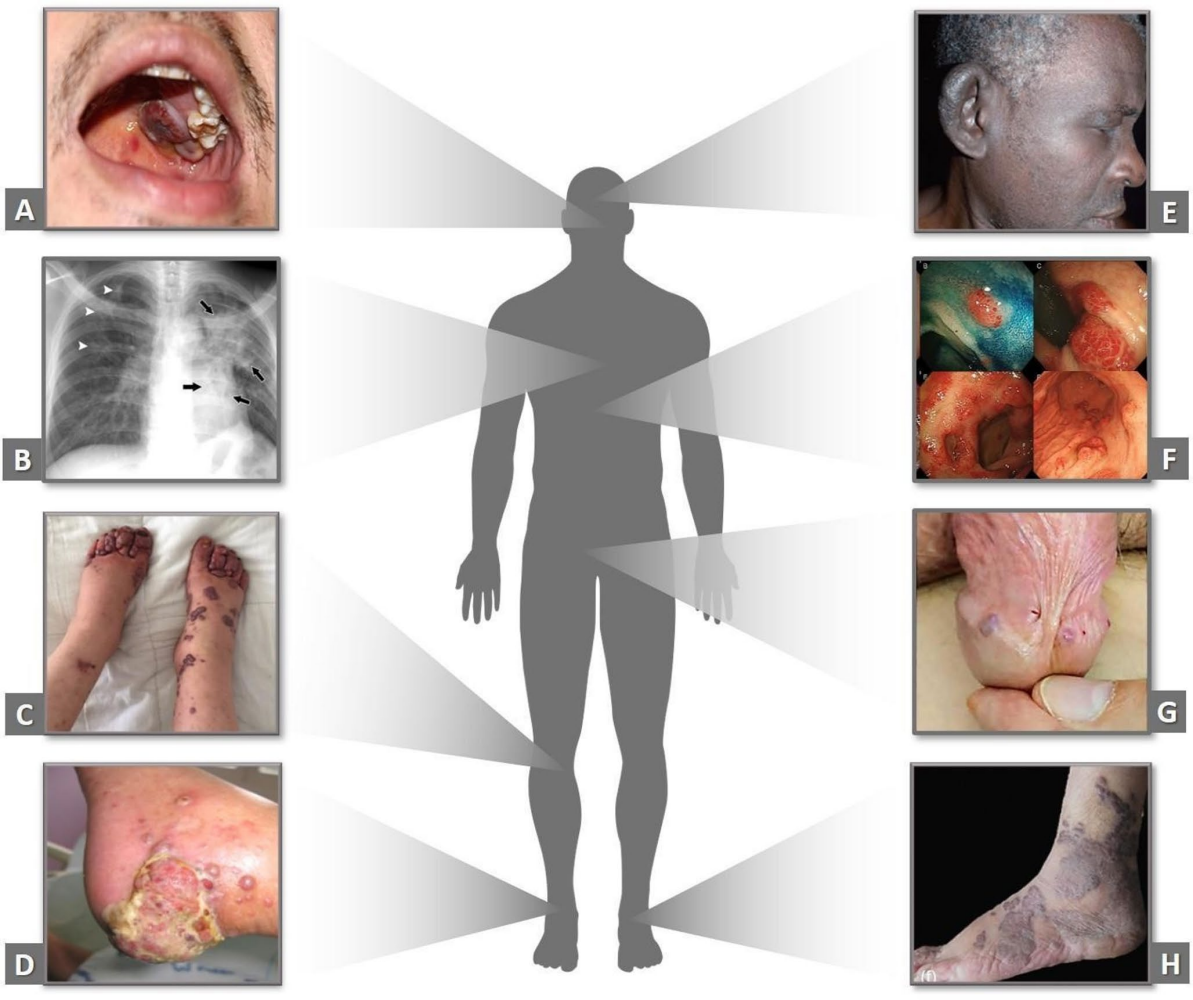
Clinical manifestations of Kaposi’s sarcoma across different anatomical sites and epidemiological forms. (**A**) Oral Kaposi’s sarcoma in an HIV-positive patient, showing violaceous nodular and plaque lesions on the hard palate. (**B**) Pulmonary involvement in an HIV-positive patient with KS, detected by chest X-ray, showing bilateral nodular and mass-like lesions (arrows). (**C**) Angiomatous cutaneous nodules associated with lymphedema in a patient with IKS. (**D**) Advanced cutaneous KS with tumor expansion and surrounding nodules in the heel of a patient with AKS. (**E**) Diffuse tumor infiltration of the external ear in a patient with AEKS. (**F**) Visceral involvement of KS-HIV patient, demonstrated by endoscopic images of gastrointestinal tract lesions. (**G**) Genital involvement in CKS, presenting as erythematous–violaceous papules on the glans penis. (**H**) Multiple violaceous cutaneous nodules of varying sizes affecting the forefoot in CKS. Images reproduced from published sources as indicated in panels A–H:Ma J.Y. and Liu J.W., 2022 (**A**); Restreo C.S.Martinez S. et al., 2006 (**B**); Elboukhari K., Achehboune K. et al. 2020 (**C**); Chapalain M., Goldman-Lévy G. et al. 2018 (**D**); Travassos A.R., Costa J.B. et al. 2010 (**E**); Nagata N., Shimbo T. et al., 2012 (**F**); Ramos M.C., Oliveira F.B. et al., 2020 (**G**); Zhou J. Shen X.Wang, X. et al. 2021 (**H**).

KS is a multifocal angioproliferative cancer that currently has four main epidemiological groups according to clinical manifestations: classic or sporadic (CKS), endemic or African (AEKS), associated with HIV/AIDS (KS-HIV) and iatrogenic (IKS), in addition to the highly aggressive anaplastic form associated with relapse (AKS) (Buchbinder and Friedman-Kien 1991; Chapalain et al. 2018; Cesarman et al. 2019). The clinical and histological manifestations shared among all epidemiological forms include well-delimited skin lesions with pigmented macules that can vary in size, depth and color (from rosy to intense violet) (Fig.2C, G, H). During tumor progression, there is an amplification of vascular fusiform cells along the dermis or mucous surfaces, which originates nodular lesions classified as tumors (Jenner and Boschoff 2002) (Fig.2D). These lesions can spread to other parts of the body, such as lymph nodes, digestive tract and/or lungs (https 2021).

CKS occurs mostly in men (10–15 men per woman), primarily affecting middle-aged and elderly individuals over 50 years of age (Schwartz et al. 2008; Di Lorenzo 2008). However, new cases are emerging in woman patients, with rapid clinical evolution atypical manifestations such as tumors in the mammary glands (Trujillo 2015; Qian 2019). This epidemiological form has a higher incidence in the Middle East, Eastern Europe and Mediterranean. However, in South America > 250 cases of CKS have been reported, including in Colombia (Iscovich et al. 1998, 1998; Mohanna et al. 2005). The predominant manifestations of CKS appear as vascular lesions and presence of larger tumor nodules that can be symptomatic, especially those located in the lower limbs, allowing the formation of ulcers and susceptibility to secondary infections. A retrospective analysis with 156 patients showed important results in the clinical outcome of patients, such as the most frequent location of the tumor in the lower limbs (92%) and the development of the tumor in the lung and liver in ~ 6.41% of patients and bone metastasis in 1.28% (Brambilla et al. 2003; Cetin 2018). In this study, according to location, distribution and progression, CKS was staged as: stage I (small macules and delimited nodules 48.1% of patients); stage II (plaques mainly on the extremities of the lower limbs in 22.4%); stage III (multiple plaques, angiomatous nodules and ulcers in the lower limbs in 19 0.9%); and stage IV (multiple plaques, angiomatous nodules extending beyond the lower limbs in 9.6%) (Brambilla et al. 2003).

AEKS is the endemic form that occurs commonly in sub-Saharan Africa in HIV negative people, affecting mainly young adult men and children of both sexes. This form remains prevalent and among the ten most common cancers in Zambia, for example, despite the availability of ART. The simultaneous occurrence of KS and HIV infection continues to pose a significant public health concern in these regions (Mankuce et al. 2025). AEKS is associated with accumulation of lymphatic fluid in adipose tissue causing edema in the legs and arms and in most cases has a poor prognosis, resulting in death within a few years (Friedman-Kien and Saltzman 1990). In AEKS, the involvement of the gastrointestinal tract and the appearance of pulmonary lesions lead to clinical worsening of the patient, such as breathing difficulties and blood expectoration (hemoptysis), which can lead to death (Friedman-Kien and Saltzman 1990; Mohamed et al. 2022) (Fig.2B, F).

KS-HIV is the epidemic form of KS associated with HIV/AIDS that was characterized during the 1990 s, when the virus was found in > 90% of KS lesions in HIV positive patients (Chang et al. 1995). On the African continent, the AEKS and KS-HIV forms represent 9% and 4% of all malignant pathologies in the regions of Zaire and Uganda, respectively (Forae and Obaseki 2018). The progression of the HIV epidemic led to the fusion of AEKS with KS-HIV, for example, vascular tumor analysis in 79 patients in Nigeria showed that out of 95.2% of KS cases 96.2% were in HIV positive patients with active antiretroviral therapy (Forae and Obaseki 2018). Among the clinical manifestations, palate and gingival lesions are characteristic of KS-HIV (Fig.2A). Clinical-laboratory study with 22 patients with HIV-KS up to 40 years old revealed that 90% of them had cutaneous lesions, 50% had visceral involvement, 86.4% had high-risk staging and 36.5% died (Lima et al. 2017). Other studies revealed that the skin lesions identified in 1,428 and 51 patients, represented 78% and 70% of them, respectively, and were violaceous macules and papules (solid lesions). Visceral injuries, including stomach, were 55% and 62.7% (Fig.2F), in addition to liver and colon injuries (Pires 2018; Silva et al. 2016). 20% of these KS-HIV patients died after aggressive tumor development followed by severe anemia and sepsis (Vally 2020).

IKS is the form of KS associated with immunosuppression that occurs, for example, after solid organ allotransplantation (Buonaguro et al. 2003)—particularly kidney transplantation—and has also been reported in recipients of hematopoietic stem cell transplants (Cesaro et al. 2020). Although rare, the incidence of KS increases 200-fold in solid organ transplant recipients (Grulich and Vajdic 2015), where in most cases it manifests in response to KSHV reactivation (Baykal et al. 2019). Prevalence risk is higher in multi-organ transplants and in cases of incompatibility; IKS can be seen in patients using immunosuppressive drugs such as corticosteroids, methylprednisolone, mycophenolate mofetil, prednisone, tacrolimus and cyclosporine (Belabbes et al. 2024; Raedemaeker et al. 2019; Frangiamore et al. 2021). Studies with 1,389 patients with KS in Italy showed that of the 143 IKS, corticosteroids and cyclosporine represented the most frequent immunosuppressants among them, classic lesions were present in 66.7% of the individuals, viscera in 9.1% and accumulation of lymphatic fluid (lymphedema) in 100%, representing a state of complication (Brambilla et al. 2017). Recent research with the SK variation showed that among 137 patients, the CKS form was the most frequent, followed by IKS in most transplanted patients (Buonaguro et al. 2003). In patients with IKS, all may have disseminated painless skin lesions or on the upper and lower extremities, ears, penis, face, cervical region and some extra cutaneous lesions such as the gastrointestinal tract, urethra and oral mucosa, however, there are cases that the cutaneous manifestation was not identified, only the visceral location (Baykal et al. 2019; Belabbes et al. 2024; Frangiamore et al. 2021) (Fig.2).

In addition, AKS represents a rare and highly aggressive variant characterized by deeply invasive vascular tumors, marked cytological atypia, and a strong metastatic potential (Chapalain et al. 2018). AKS often arises in the context of prior KS, suggesting progression from less aggressive forms, and frequently requires radical therapeutic intervention (Fischer et al. 2023) (Fig.2).

## KSHV transmission

Initially, the sexual route was described as the main route of viral transmission and considered a cofactor for the evolution of KS (Jeffrey et al. 1998; Beral et al. 1990). However, subsequent studies have suggested that sexual transmission is not essential for KSHV infection, as high prevalence rates are observed in endemic and hyperendemic regions (Biggar et al. 2000; Cunha et al. 2005; Plancoulaine et al. 2000). Sexual transmission was first described in male patients who have sex with men (Gottlieb 1981). Data from the literature have reported the detection of viral DNA in the prostate, seminal fluid, and spermatozoa of patients coinfected with HIV and KSHV (Howard et al. 1997; Monini et al. 1996; Bagasra et al. 2006). Studies through PCR analysis of semen samples from 100 patients with HIV showed the detection of KSHV DNA in 26% of those analyzed (Smith 1999). In addition to sexual transmission and co-infection, injecting drug use also represents a risk factor for KSHV infection (Cannon et al. 2001; Alamri and Adiga 2019). A cohort study of women showed the detection of anti-KSHV antibodies in vaginal and cervical specimens from both HIV-positive and HIV-negative individuals (Lampinen et al. 2000).

Among body fluids, saliva is the site of greatest detection of high viral concentrations, which suggests the replication of KSHV in tonsils with distribution in saliva (França et al. 2011; Casper et al. 2007; Aalam et al. 2020). Viral transmission through blood is still not well characterized, as there is inconsistency in the various studies on this route (Yan et al. 2019). However, studies showed the presence of the virus in the blood tissue of patients infected with KSHV, which can lead to transfusional transmission (Hladik et al. 2006; Vamvakas 2010). Although rare, there are reports of high seroprevalence in patients with KS who received KSHV-contaminated blood: 36.2% in 1,761 patients (Hladik et al. 2006), > 40% in 174 patients (Enbom et al. 2002)and 55% in 11 patients (Whitby et al. 1995). In mucosal regions, identification of KSHV is more frequent in the oropharynx and orolabial region (Casper et al. 2007; Atyeo et al. 2021). By investigating individual, household, and regional factors influencing KSHV reactivation and oral shedding, 149 out of 340 participants (44%) were identified as “shedders”—that is, they shed KSHV orally at least once (Sabourin et al. 2024).

Organ transplantation represents an additional route of viral transmission, and a markedly increased incidence of KS has been reported among organ transplant recipients (OTRs), with rates up to 200-fold higher than in the general population. KSHV infection associated with transplanted grafts remains underreported, largely due to limitations in diagnostic screening and viral monitoring (Pria 2025).Genetic analysis of cells from KS lesions in transplant patients revealed differences in the genetic profile of these cells, indicating that viral transmission can occur from donor cells (Barozzi et al. 2003). Recently, a study carried out with 22 organ donors positive for KSHV revealed viral infection in 64% of recipients, of whom 6 developed KS and 4 died (Dollard 2021). In a study in the Middle East with 263 patients undergoing transplantation, KS was diagnosed in 5.3% of cases after 29 to 31 months of transplantation (Montagnino et al. 1994; Parravicini et al. 1997). Post-transplant immunosuppressive therapy contributes to viral dissemination and reactivation, thereby increasing cancer risk. An increase in anti-KSHV antibody detection was observed from 6.4% before transplantation to 17.7% one year after renal transplantation in a cohort of 25 patients, with two individuals developing sarcoma 26 months after surgery (Regamey et al. 1998). Even before transplantation, a follow-up study of 100 patients awaiting renal transplantation with immunosuppressive therapy revealed an increase from 12% to 26% in the seroprevalence of these patients over a 9-year period (Cattani et al. 2001).

## KSHV intracellular cycle

**Fig. 3:**
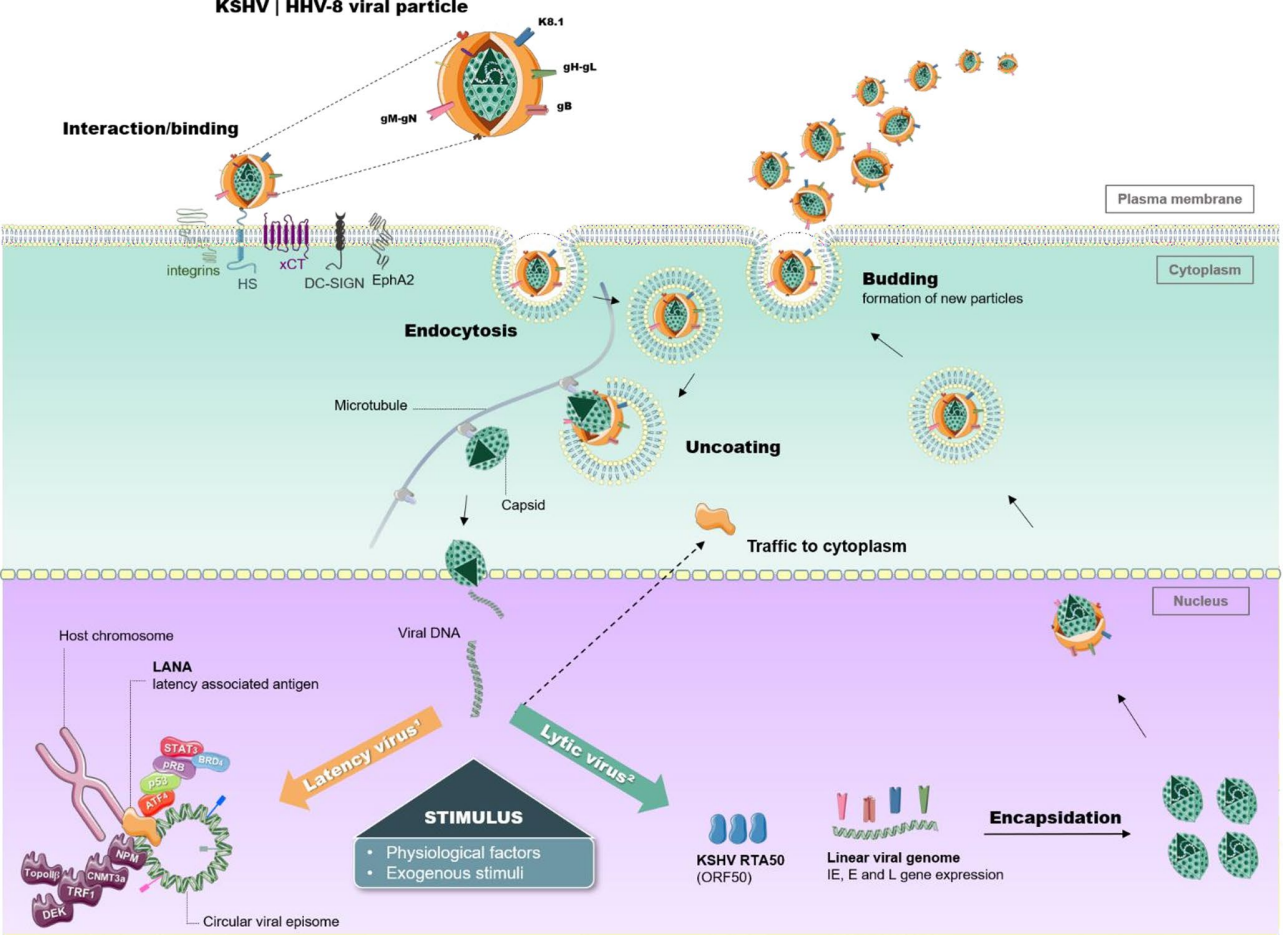
Cell invasion and KSHV cycle. After binding to host cell surface receptors, KSHV enters target cells through interactions between viral envelope glycoproteins—including gpK8.1, gB, gM–gN, and the gH/gL complex—and cellular receptors such as heparan sulfate (HS), EphA2, and xCT. Viral entry occurs predominantly via endocytosis, followed by capsid transport along microtubules and delivery of the viral genome into the nucleus. Once in the nucleus, KSHV establishes latency as a circular episomal genome tethered to host chromatin by the latency-associated nuclear antigen (LANA), or alternatively enters the lytic replication cycle upon stimulation, characterized by activation of the replication and transcription activator (RTA), viral genome linearization, and sequential expression of immediate-early (IE), early (E), and late (L) genes, culminating in virion assembly and release. During latency, LANA interacts with host transcriptional, tumor suppressor, and epigenetic regulators to maintain episomal persistence and repress lytic gene expression while preserving the potential for reactivation. *HS* heparan sulfate, *EphA2* ephrin receptor A2, *xCT* cystine/glutamate transporter, *IE* immediate-early genes, *E* early genes, *L* late genes. Some elements of the illustration were created using Servier Medical Art

In the process of viral transmission, KSHV cell invasion occurs through interaction between viral envelope glycoproteins and cell surface molecules (Yan et al. 2019; James 2004). Glycoprotein-B (gB), glycoprotein H (gH), glycoprotein L (gL) - common to other components of the Herpesviridae family, and K8.1 (expressed only in KSHV) are considered essential for virus-host interaction (Veettil et al. 2014; Dollery and Towards Understanding 2019) (Fig.3). In the host cell, surface proteoglycans such as heparan sulfate (HSPGs) participate as receptors for viral adsorption, binding to the viral ligands gB, the functional complex gHgL, and K8 (Dollery and Towards Understanding 2019). In the absence of glycosaminoglycans (GAG), alternatively KSHV binds to cell surface α3β1, α3β3 and α3β5 integrins, abundant in B cells (Kerur et al. 2010; Nemerow and Cheresh 2002; Veettil et al. 2008) (Fig.3).

The cystine/glutamate transporter (xCT) participates as a fusion receptor integrated into the integrin signaling complex, and can be upregulated by KSHV genes to amplify the susceptibility of endothelial cells, macrophages, BC-1, BCBL- 1 and BCP-1 (Kaleeba and Berger 2006; Dai et al. 2014). The DC-SIGN lectin receptors present on DC’s and macrophages may function as adhesion molecules and favor viral infection (Kerur et al. 2010; Garcia-Vallejo and van Kooyk 2013). In addition, the type A ephrin receptor 2 (EphA2) acts as a central point in the processes of signaling and regulation of endocytosis in several cell types (Fig.3). Literature data demonstrated that the knockdown of EphA2 is related to the attenuation of the infection, while its overexpression increased viral entry (Dollery and Towards Understanding 2019; Hahn et al. 2012). KSHV enters the cell by endocytosis and translocates to the nucleus, followed by virus replication, editing and construction of various viral particles, characterizing the lytic phase (Yan et al. 2019; Bu 2008). DNA becomes linear to express the entire viral repertoire starting with immediate-onset genes (That is, genes expressed immediately after viral reactivation and do not require pre-synthesis of KSHV proteins) (Zhu 1999). The lytic cycle initiates the replicative process in regions of ori-Lyt (L) and ori-Lyt (R) functional origins, with the involvement of cellular and viral components. During lytic replication, the structural genes of the capsid, envelope and tegument are expressed for the formation of new particles through steps of, integration of the genetic material into the capsid; encapsulation of the capsid by the envelope in the nucleus for release into the cytoplasm; release of viral particles after fusion of the vesicle membrane with the cell membrane (Dollery and Towards Understanding 2019) (Fig.3).

After a short lytic phase, the intracellular cycle evolves into the predominant latent phase. During latency, the KSHV genome adopts a circular, extrachromosomal episomal conformation and is stably maintained through replication and segregation during mitosis (Hu et al. 2002). The latent cycle is characterized by the expression of only a limited set of latency-associated genes involved in the establishment of a successful long-term infection. Among these, ORF73 encodes LANA (latency-associated nuclear antigen), a homolog of EBV-encoded EBNA1, which is a key oncoprotein in the modulation of KSHV infection (Wei and Gan 2016), as it is essential for maintaining viral episomal persistence (Fig.3). Moreover, the KSHV episome undergoes extensive epigenetic modifications, including DNA methylation and histone acetylation, which regulate the chromatin state during infection. The viral genome displays a highly structured spatiotemporal organization. This organization involves the formation of genomic loops that enable coordinated control of dynamic gene transcription. This epigenomic pattern is not static: extracellular conditions such as hypoxia and inflammation directly influence these epigenetic marks, modulating the architecture of viral chromatin and, consequently, the expression of KSHV genes (Inagaki and Kumar 2024).

For example, LANA interacts with the tumor suppressor p53 (p53) and represses its transcriptional activity, inhibiting its ability to induce cell death (Vamvakas 2010; Chen 2010). In line with these observations, KSHV can be reactivated in response to several conditions such as hypoxia, treatment with chemical agents, and host cell molecular events (Davis et al. 2001; Yu et al. 1999; Balistreri 2016). During reactivation, LANA dissociates from chromatin allowing its translocation from the nucleus to the cytoplasm (Sharon et al. 2018). It is important to highlight that the switch for the lytic phase is controlled by RTA50 (ORF50), a fundamental regulatory protein for virus reactivation, which could be modulated by miRNAs (Bu 2008). The repression or activation of ORF50 depends on multiple regulatory mechanisms. These include interactions with cellular proteins that modulate KSHV transcription (Bu 2008; Lin et al. 2022), self-regulation via association with the transcription factor octamer-binding protein 1 (Oct-1) (Sakakibara et al. 2001), and interaction of RTA50 with the CCAAT-enhancer binding protein α (C/EBPα) (Wang et al. 2003). Thus, the balance between latent and lytic gene expression is a key determinant of KSHV pathogenesis.

## The KSHV and hallmarks of cancer

Cancer, in general, has biological attributes acquired throughout the development processes for its formation. These elements constitute an organizational regimen to support the complexities of neoplasms. This principle includes attributes that involve sustaining proliferative signaling, preventing growth suppressors, resisting cell death, allowing replicative immortality, inducing angiogenesis, activating invasion and metastasis, evading immunological destruction, among others (Hanahan 2022).

Recent evidence on viral oncogenesis indicates that oncogenic viruses establish malignancy through coordinated epigenomic, transcriptional, and metabolic reprogramming. Viral infections are responsible for approximately 1.4 million new cancer cases worldwide each year, accounting for about 8% of all human tumors (Xiao et al. 2025). Mechanistically, oncogenic viruses remodel the host chromatin landscape through the selective enrichment of activating histone marks (H3K4me3, H3K27ac) and repressive marks (H3K27me3, H3K9me3), accompanied by DNA methylation changes that stabilize transcriptional programs associated with latency. Viral proteins and ncRNAs further sustain chronic activation of oncogenic signaling pathways—including PI3K/AKT/mTOR, MAPK/ERK, JAK/STAT3, and NF-κB—thereby promoting cell proliferation, inflammation, and resistance to apoptosis (Xiao et al. 2025). These alterations are complemented by metabolic adjustments such as increased glycolysis, glutaminolysis, and lipid biosynthesis, which support the energetic and biosynthetic demands of infected cells. Together, this integrated model demonstrates how viruses such as KSHV exploit convergent regulatory nodes to promote persistence and tumorigenicity (Xiao et al. 2025).

**Fig. 4:**
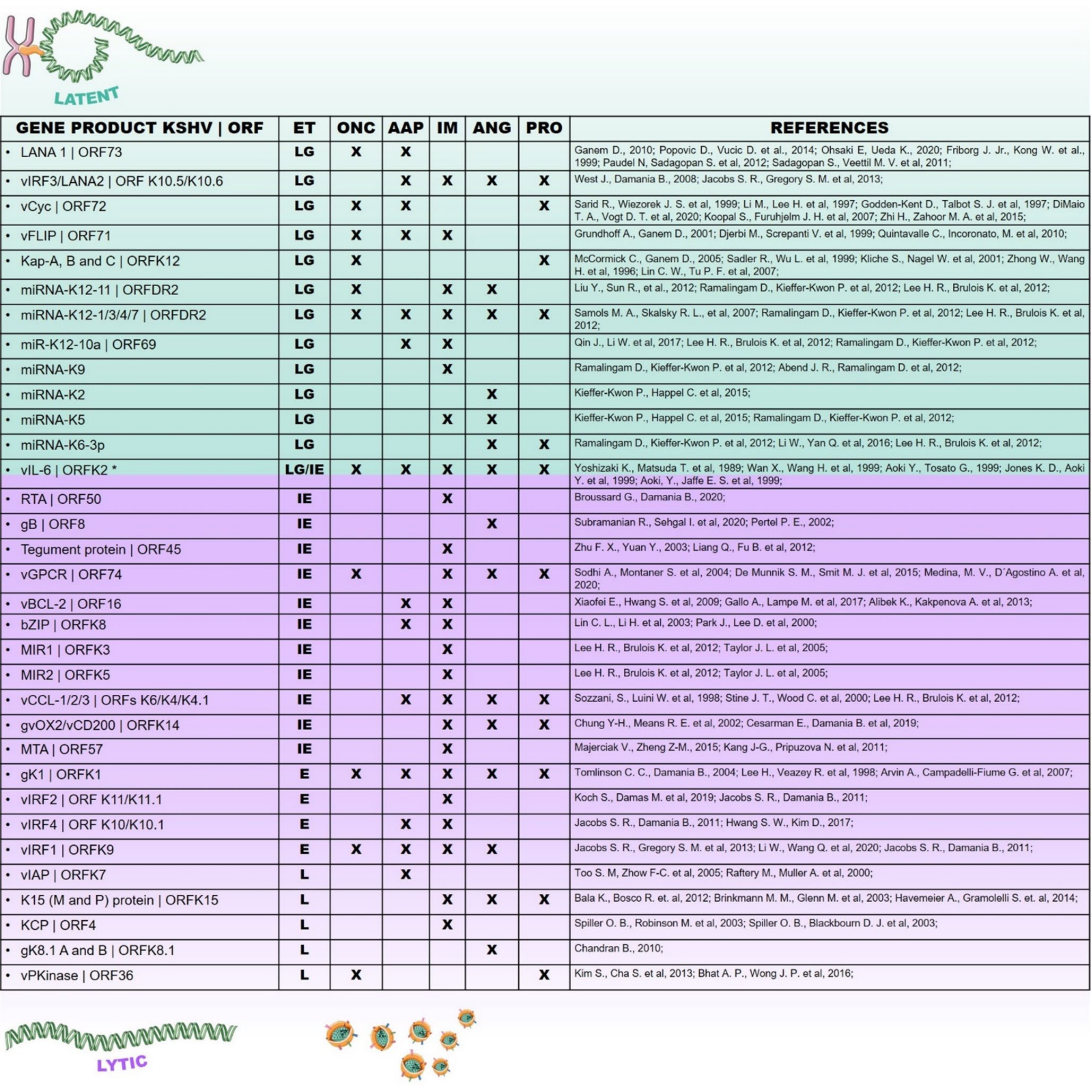
KSHV gene products expressed in latent and lytic cycles, and involvement in tumorigenic mechanisms. The list on the left shows a group of genes/products expressed during the latent low-productivity state. On the right, during the viral lytic phase, KSHV expresses its complete gene repertoire, including those presented here, enabling the formation of new viral particles. *ET* expression time, *ONC* oncogenesis, *AAP* anti-apoptotic, *IM* immunomodulatory, *ANG* angiogenic, *PRO* pro-inflammatory, *LG*, latent gene, *IE* immediate onset gene, *L* late gene, *E* delayed early gene, *ORF* open reading frame; * The vIL-6 gene can be expressed during viral latency (LG) and/or immediate onset (IE); v, viral; g, glycoprotein. Some parts of the illustration were constructed using images from Servier Medical Art

**Fig. 5:**
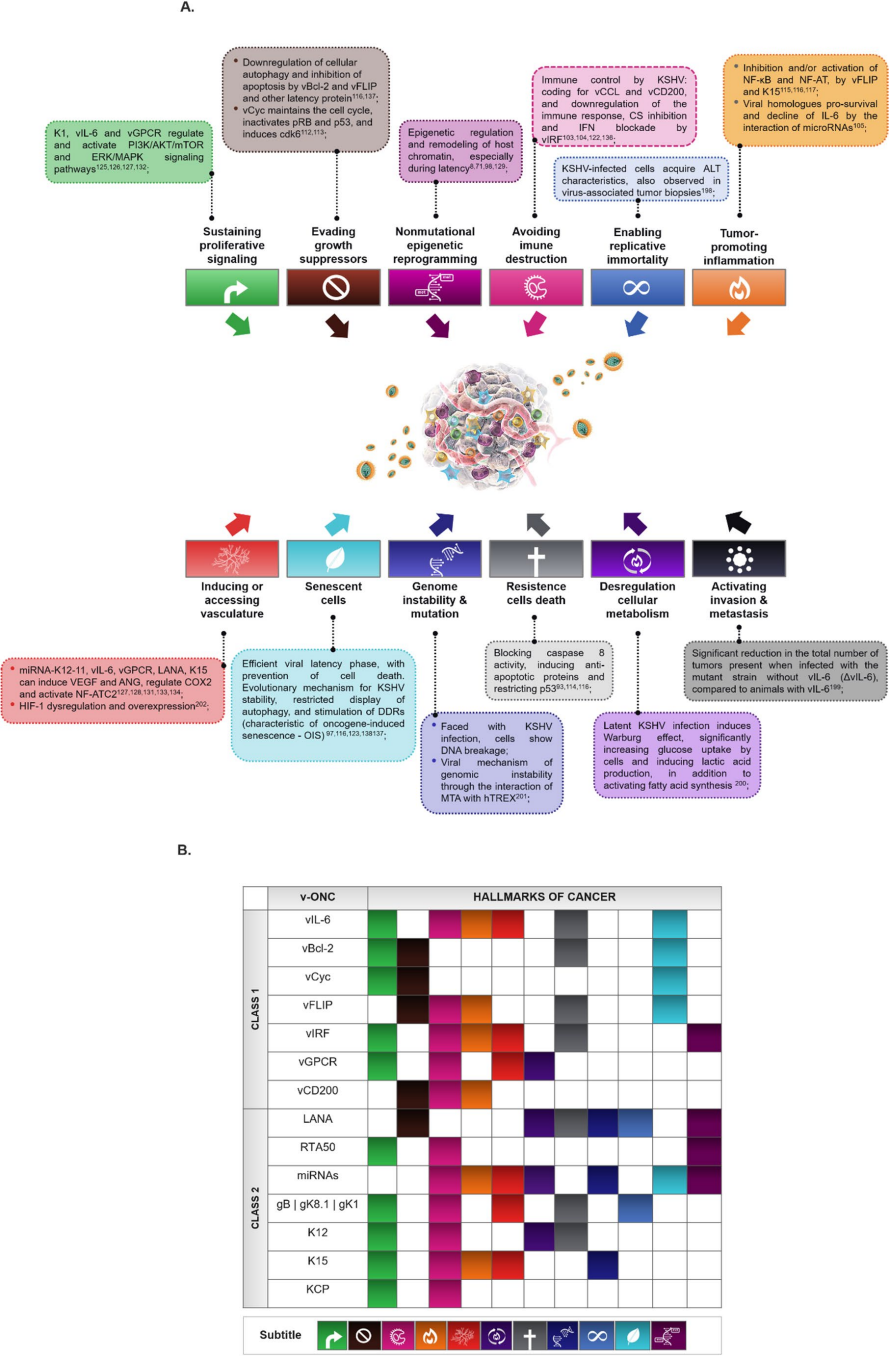
KSHV oncogenic processes and molecules associated with the Hallmarks of Cancer. InitiallyHanahan and Weinberg proposed an ordered set of ten traits that characterize tumor development, as the understanding of cancer mechanisms evolved, other facilitating and debateable aspects emerged and were included by Hanahan D., 2022 adding then 14 brands. KSHV has numerous cell transformation mechanisms, and it was analyzed to evaluate all these characteristics, totaling regulate/enable 12 of the 14 cancer promotion brands (**A**). Here, some of these fundamental viral oncogenic mechanisms are categorized for each hallmark of cancer. (**B**) The figure lists some oncogenic viral molecules (v-ONC), fundamental to tumorigenic processes, which contribute to the characteristics of cancer. MTA, ORF57; hTREX – cellular transcription and export complex. Some parts of the illustration were constructed using images from Servier Medical Art

The oncogenic molecular machinery of KSHV is highly complex and efficient, which makes this virus a determining agent for tumor development, while the viral infection coexists in the individual. In addition, the integrity of the immune system is an indisputable aspect for oncogenic viral factors to play their tumorigenic role. Currently, there are 14 characteristics of cancer described in the literature (Hanahan 2022) and KSHV may be involved in 12 of them, such as (1) support for proliferative signaling; (2) evasion of tumor suppressors; (3) non-mutational epigenetic reprogramming; (4) immune evasion; (5) enabling replicative immortality; (6) tumor-promoting inflammation; (7) activation of invasion and metastasis; (8) vascularization; (9) cellular senescence; (10) genome instability and mutation; 11) resistance to cell death; and 12) reprogramming of cellular metabolism (Fig.5). To support the participation of the virus in these tumor processes, KSHV has the ability to express two classes of oncogenic molecules described here as: (i) CLASS 1 (functional homologues - FH), mainly regulatory and cell signaling proteins, such as vIL-6 (ORF K2), vBcl-2 (ORF 16), vCyc (ORF72), vFLIP (ORF 71 or ORFK13), vIRF1 (ORF K9)/vIRF 3 (ORF K10.5, ORF K10.6), vGPCR (ORF 74) and vCD200 (Lopes et al. 2022; Shiratori 2005), and (ii) CLASS 2 (non-homologous gene products - NHGP), such as LANA (ORF73), RTA50, miRNAs, glycoproteins, K12, K1 (ORF K1), K15 (ORF K15), KCP and vPKinase (ORF 36) (Bu 2008; Lopes et al. 2022; Qin et al. 2017; Subramanian et al. 2010; Spiller et al. 2003) (Figs.4 and 5B).

## Oncoproteins associated with latency

It is possible to classify the oncogenes involved in lytic and latent cycle replication by their organization in the DNA and by transcriptional and translational activity during the viral cycle. The genes of the latent replication program are classified as latent gene (LG). LANA (ORF73) is the main latent oncoprotein responsible for activating oncogenesis pathways, for example, through repression and inactivation of p53 (Friborg et al. 1999; Chen 2010); inactivation of GSK3 β – a serine threonine kinase, to promote cell proliferation; activation of the c-Myc oncoprotein, through ERK (Liu et al. 2007, 2007); induction of ribonuclease 5/angiogenin (ANG, angiogenic stimulator), which blocked, inhibits latency genes and promotes viral reactivation and inactivation of retinoblastoma tumor suppressor protein (pRB) (Paudel et al. 2012; Radkov et al. 2000) (Figs.4 and 5B).

As well as LANA, functional viral cyclin (vCyc- ORF72) belongs to the latency cluster and presents functional and structural similarity to its human homolog cyclin D (Li et al. 1997). vCyc binds and activates cyclin-dependent kinase 4/6 (Cdk’s 4/6) maintaining the cell cycle of the infected cell and inactivating the pRB (Li et al. 1997; Godden-Kent et al. 1997). The oncogenic potential of viral cyclin expressed by KSHV has already been evidenced by p53 suppression in vivo (Verschuren et al. 2002).

Viral FADD-like interleukin-1-β-converting enzyme inhibitor protein [FLICE/caspase 8] (vFLIP-ORF 71), a homologous protein of cellular FLIP (cFLIP), is expressed during latent infection and has been shown to be essential for the survival of infected tumor cells due to antiapoptotic functions, autophagy suppression and NF-κB activation (Grossmann et al. 2006; Lee et al. 2009) (Figs.4 and 5B). The NF-κB complex activates the transcription of inflammatory cytokine genes, in addition to mobilizing the Wilms tumor protein - WT1 (already reported to be overexpressed in KS lesions and other tumors), which creates a favorable microenvironment for tumor development (Morales 2021). Furthermore, vFLIP has been described to induce morphological change of endothelial cells into fusiform and elongated, characteristic of KS lesions (Alkharsah 2011). Most of these cells in the areas of lesion present the latent form of infection, but a portion of them with lytic replication can also be seem (Zhong et al. 1996).

## Oncoproteins associated with the lytic cycle

During the lytic cycle, the production of new viral particles and the expression of oncogenic factors are predominant, which contribute to an angiogenic and inflammatory phenotype. The different phases of gene expression during the lytic cycle are classified as IE- immediate early/immediate onset genes (expression independent of viral protein synthesis) (Zhu 1999; Wang et al. 2003; Wang 2004; Saveliev et al. 2002); DE or E- delayed early genes (expression independent of viral DNA synthesis) (Koch 2019)and L- late genes (expression of genes encoding capsid proteins and envelope glycoproteins dependent on KSHV DNA synthesis) (Li et al. 2018; Lu 2004) (Fig.4). Cells infected in the lytic cycle express proteins such as K1 (ORF K1), vIL-6 (ORF K2) and vGPCR (ORF 74) which, especially in endothelial cells, activate the PI3K/AKT/mTOR signaling pathway, regulating the cell cycle by inhibiting apoptosis and activating cell proliferation (Montaner et al. 2003; Tomlinson et al. 2004; Morris et al. 2008). The homologous functional protein vIL-6 interacts with the cellular transmembrane protein gp130 to increase vascular permeability by inducing vascular endothelial growth factor (VEGF) (Morris et al. 2008; Sakakibara and Tosato 2011); promote cell proliferation (Han et al. 2024); induce angiogenesis and tumorigenesis by epigenetic regulation (Han et al. 2024); deregulate the NF-κB signaling pathway, a transcription factor involved in several pathophysiological processes of cancer (Charostad et al. 2020). The PI3K/AKT/mTOR and NF-kB signaling pathways are also regulated by vGPCR, a viral G protein-coupled receptor homologous to the IL-8 angiogenic receptor, that exerts the function of modulating angiogenic molecules such as TNF-α and VEGF and increasing secretion of interleukins (De Munnik et al. 2015). The ERK/MAPK signaling pathway is also triggered by vGPCR to upregulate prostaglandin H2 synthase (COX2), a crucial molecule in cancer angiogenesis (Medina et al. 2020).

The K15 (ORF K15) non-structural viral membrane protein participates in the activation of NF-ATC2, a member of the NF-AT family, thereby promoting angiogenesis through capillary formation, and also contributes to the suppression of apoptosis by activating the Akt pathway (Bala 2012; Brinkmann et al. 2007; Brinkmann et al. 2003). The viral interferon regulatory factors 1 and − 3 (IRF-1/ORF K9 and IRF-3/ORF K10.5, ORF K10.6) are homologues of the cellular IRF family that negatively regulate interferon (IFN) gene transcription and additionally have the potential to repress type I IFN signaling via toll-like receptor 3 (TLR3) (West and Damania 2008; Jacobs et al. 2013). The viral homologue vBcl-2 (ORF16) regulates apoptotic cell death and may facilitate escape from cellular autophagy (Xiaofei et al. 2009); moreover, vBcl-2 is considered indispensable for viral reactivation, influencing DNA replication and the formation of new viral particles (Gelgor et al. 2015). The glycoproteins B (gB) and K8.1 also play a role in endothelial cell transformation and angiogenesis (Purushothaman 2016). Finally, the T0.7 transcript (ORF K12) is an oncogene that encodes the kaposin protein (K12) (Sadler et al. 1999), which is involved in the cellular transformation process associated with tumor development and inflammatory cytokine expression; this protein is detected across all epidemiological forms of KS and in BCBL-1, PEL, BC-3, and KS-1 cell lines (Staskus et al. 1997; Renne et al. 1996; Muralidhar et al. 2000, 1998) (Figs.4 and 5B).

## miRNAs

The 12 pre-miRNAs and 25 mature miRNAs have been identified within the genome of KSHV (Catrina 2014), make up the CLASS 2 molecules and could produce the expression of effective cell gene that favor virus maintenance and cancer development. After interacting with their respective cellular targets could: (i) maintain KSHV latency, (ii) enhance angiogenesis and dissemination of the infected cells, (iii) regulate host gene expression for tumor promotion, (ii) enable the escape of the immune system by regulating viral and cellular gene expression and (iii) protect infected cells from the effects of infection-mediated DNA damage (such as cell cycle arrest and apoptosis) (Catrina 2014; Liu et al. 2017; Ramalingam and Ziegelbauer 2017).

Among the miRNA involved in cellular modulation, the miRNA-K12-11 downmodulates TGF-β signaling through Smad-5 suppression, facilitating cell proliferation. Other miRNAs, such as BART-5, BHRF1-3, miR-K12-1/−3/−4/−7, miR-K12-10a, have their cellular targets already identified and are described to induce: (i) resistance to apoptosis; (ii) evasion of the immune system; (iii) tumor progression; (iv) maintenance of viral latency; (v) transcriptional reprogramming; (vi) reduction of IL-6 expression; (vii) reduction in TGF-β activity (Samols et al. 2007; Skalsky et al. 2007; Nachmani et al. 2009; Choi et al. 2015; Abend 2012; Ramalingam et al. 2012). MiR-375 was identified as a potential marker of active KS (Piano et al. 2019). It was also described that KSHV miRNAs could suppress several targets associated with STAT3, deregulating cytokine-mediated gene activation, inhibiting interferon response, thus influencing the transition into the lytic phase of viral replication (Ramalingam and Ziegelbauer 2017) (Figs.4 and 5B). Furthermore, the presence of exclusive KSHV miRNAs, such as miR-155, miR-222/−221 and miR-143/−145, could help to elucidate the tumor tissue origin, stage and proliferative phase, not only in KS, but also in other DLPs (O’Hara et al. 2009).

Furthermore, three viral miRNAs were identified (miR-K12-6-3p, miR-K12-7-3p, and miR-K12-11-3p) which act as inhibitors of the cell’s cytosolic DNA-sensing pathway, the cGAS/STING pathway, during latency. They bind directly to STING1 mRNA, repressing its translation. As a result, STING protein levels decrease, and activation of the cGAS/STING pathway in response to stimuli (such as agonists) is attenuated. Conversely, when these miRNAs are removed from the viral genome (miRNA-deletion mutants), infected cells show increased STING levels, and KSHV lytic reactivation is significantly delayed—with reduced expression of lytic genes and lower production of viral particles. This reveals a sophisticated innate immune evasion strategy by the virus: by using its own miRNAs to repress STING, KSHV silences a key viral DNA-sensing pathway, preventing antiviral activation during latency (Paulsen et al. 2025).

With its oncogenic etiological competence in KS and in the development of other DLPs, KSHV was described to present an arsenal of gene products and viral components (some discussed here), which establishes a direct connection, whether in the regulation and/or activation of some of the hallmarks of cancer (Fig. 5).

## Classic and contemporary KS diagnosis and treatments

### KS diagnosis KS diagnostics and KSHV detection

**Fig. 6:**
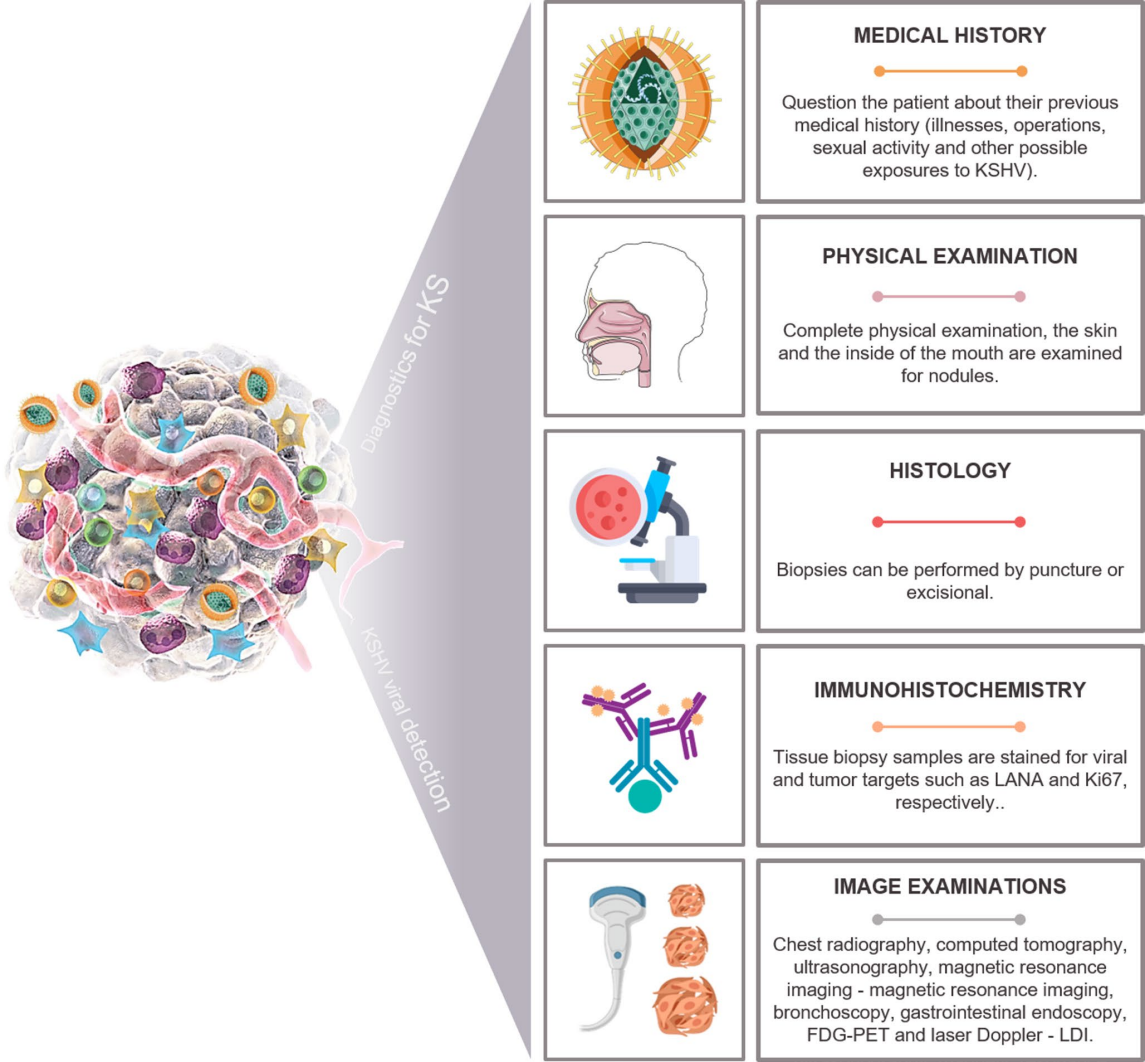
Main diagnostic methods used in clinical practice to detect KSHV and KS. Representative scheme of the main methods currently used to diagnose KS. Some parts of the illustration were constructed using images from Servier Medical Art

In a broad clinical context, KS is often diagnosed when patients seek medical care due to the presence of signs or symptoms. On the other hand, cancer can be detected during routine tests. If suspected, a set of tests is required to confirm the exact KS. Thus, these diagnostic tools in clinical practice mostly include: (I) Medical history and physical examination - the patient is asked about his medical history to learn about illnesses, operations, his sexual activity and other possible exposures to KSHV. Through the complete physical examination, the skin and interior of the mouth is examined for nodules. In cases of lesions in regions such as the rectum, stool tests are performed to detect bleeding; (II) Histology and immunohistochemistry from biopsy of the lesions - these biopsies can be by puncture (removal of a small portion) or excisional (removal of the entire lesion). These samples can be stained for CD31, CD34, LANA, D2-4, Ki67, ERG, gp64, ORF45, gB, VP26 or TRI-2 and VH; (III) Imaging tests - chest X-ray, CT, ultrasound, magnetic resonance - MRI, bronchoscopy, gastrointestinal endoscopy (including colonoscopy), FDG-PET, and laser Doppler – LDI (Fig. 6).

Despite the varied histological and epidemiological forms of KS, its diagnosis is often based on the clinical suspicion of the patient, without histopathological findings. But it is important to emphasize that the method of histopathological confirmation and marking for LANA, as a diagnosis for KS, is still considered the gold standard. Cutaneous KS usually manifests itself with well-established nodular patches. However, this “classic KS scenario” can become a pitfall and lead to an incorrect or contestable diagnosis, as there are many loopholes and exceptions, such as: (1) a positive LANA stain unequivocally confirms a diagnosis for cancer in the context appropriate clinicopathological; (2) LANA markings are variable, in addition to the fact that this marker indicates only latent infection (located in the nucleus) and not lytic (localization in the cytoplasm); (3) these classic manifestations, do not occur in all groups of SK (Qian 2019; Ramos-da-Silva et al. 2006; Garrigues et al. 2017); (4) superficial dermal lesions can be minimal or punctual (Qian 2019; Alamri and Adiga 2019); (5) confusion with cutaneous manifestations associated with other systemic and vascular diseases, as the skin exposes the reflection of disorders in internal processes and organs (Sampaio et al. 2021); (6) the morphological spectrum of KS can mimic many neoplastic conditions or unassociated pathologies (Oehler et al. 1993; Teixeira et al. 2014).

To evaluate the degree of cancer dissemination, the tumor staging is measured through: (i) growth rate and the extent of the pathology, (ii) type of tumor and (iii) relationship of cancer with host. However, to date, the KS evaluation has not been encompassed or unified to the tumor, nodule, and metastasis (TNM) staging system of the American Joint Committee on Cancer (AJCC). Tagging protocols for groups such as AEKS and IKS do not exist, but on the other hand, in the case of KS-HIV, the classification is given by the AIDS Clinical Trials Group (ACTG), considering 3 factors: tumor extension; immunological condition measured by the number of CD4 cells; and systemic disease (Krown et al. 1997). In CKS, the system is classified as agreement, only in cutaneous tumors (Brambilla et al. 2003).

### Classical therapies and clinical studies for the treatment of KS

Therapeutic tools approved by the Food and Drug Administration (FDA) for the treatment of Kaposi´s sarcoma have been used for decades, however, due to the diversity of clinical-pathological manifestations of this cancer, there is a need for specific treatments directed to the various clinical scenarios.

In general, in immunosuppressed patients, first-line procedures include stimulating the immune system, especially in the case of KS-HIV patients. For individuals of the IKS group, treatment is conducted to reduce or replace immunosuppressive drugs already used, but this practice could contribute to the increased risk of graft rejection. As KS is an indolent cancer being mainly multifocal and multiregional, the typical primary therapeutic management is a systemic treatment based on chemotherapy agents, administered orally or intravenous. Systemic chemotherapy drugs most frequently used to treat KS include the liposomal anthracyclines group and paclitaxel, but other chemotherapy agents can also be used, such as gemcitabine, vinorelbine, bleomycin, vincristine, and etoposide (https 2021, 2021; Schwartz et al. 2008; Di Lorenzo 2008). As new possible treatment alternatives (Table1), in a recent search in the World Clinical Trials Database (https://clinicaltrials.gov/), we filtered studies that were exclusively interventional (a type of clinical study in which participants are assigned to groups that receive one or more interventions/treatments, or no intervention, to evaluate the effects of interventions on biomedical or health-related outcomes by drugs), obtaining a total of 115 studies targeting KS and 1 study for viral co-infections (including KSHV) in patients with active antiretroviral therapy under analysis in the World Clinical Trial database (analyzed until the year 2023). Of this total, 94 of them were directed to HIV positive and/or HIV^−^ patients, and only 10 to CKS, AEKS or HIV negative patients. Furthermore, 74 studies have been completed (21 in phase I, 3 in phase I/II, 34 in phase II, 1 in phase II/III, 9 in phase III, 2 in phase IV and 4 in phase EU); 19 studies are recruiting (6 in phase I, 2 in phase I/II and 11 in phase II); 15 studies have been withdrawn (4 in phase I, 4 in phase I/II, 5 in phase II, 2 in phase IV); 5 studies are unknown (4 in phase III and 1 reported as “not applicable”); and 3 studies are in active status, not recruiting (1 in phase I/II, 1 in phase II and 1 in phase III) (Table 1).

**Table 1:**
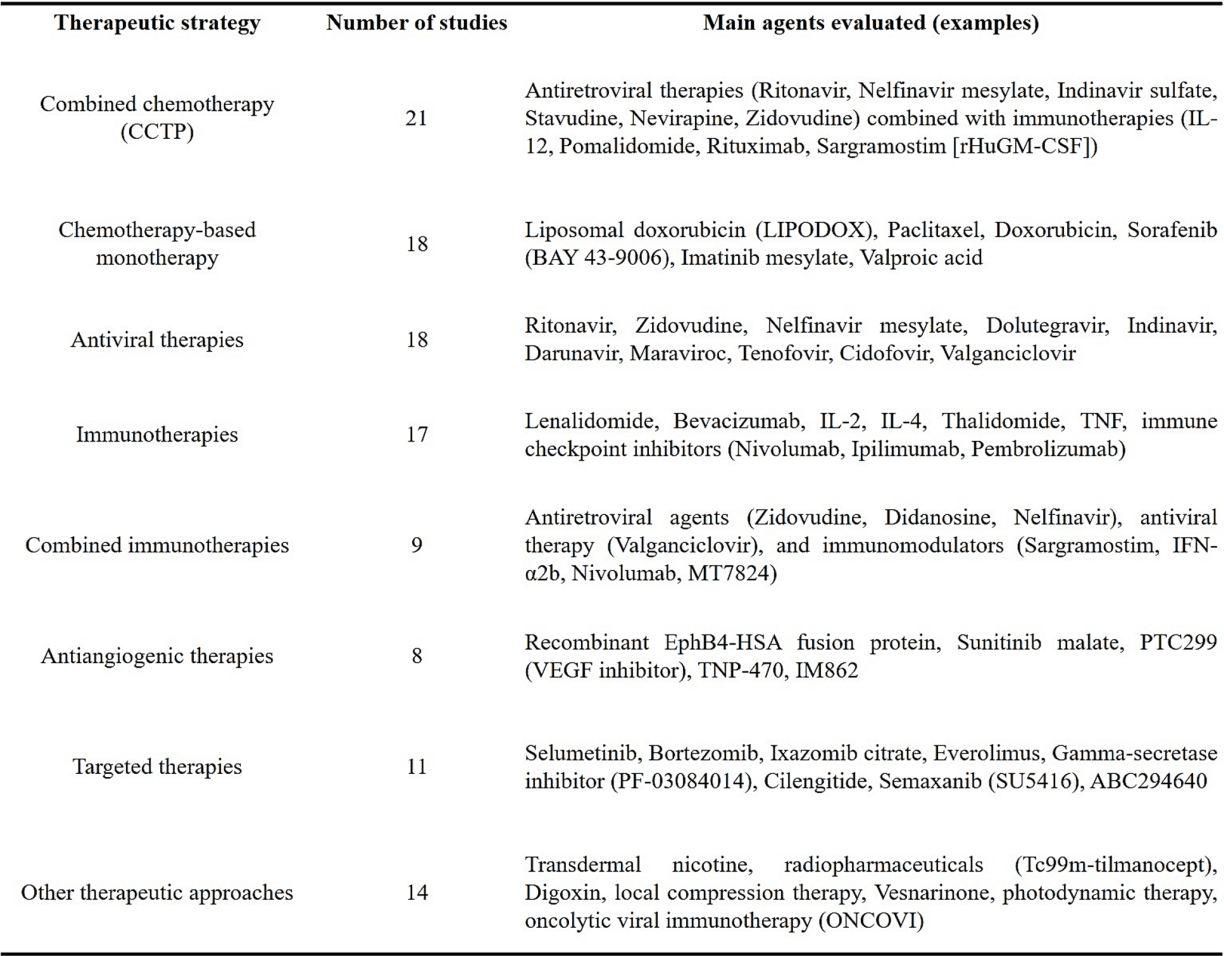
Therapeutic strategies evaluated for KS across clinical studies. Promising studies are being conducted targeting viral agents that are precursors of lymphotropic diseases, among which we can highlight:

These overall data support the fact that there is a paucity of studies designed for almost all KS groups and, although KS-HIV leads in number of studies, only 8 of 94 are in phase II of patient recruitment.

The information observed from these clinical trials is listed in Table 1.

### Antiviral drugs

Valganciclovir is an antiviral prodrug that targets viral DNA polymerase and is widely used for the treatment and prophylaxis of cytomegalovirus infection; in the context of KSHV, it has been investigated for its potential to suppress lytic viral replication (Casper 2008). Therefore, a clinical study to evaluate the effects of this drug on KSHV replication was administered orally to infected patients. After 18 weeks, by quantification of the genetic material of the virus by PCR, a 46% reduction in replication was observed by the oral route (Casper 2008). In addition to this, an in vitro study administered Valganciclovir in combination with Zidovudine (AZT - thymine nucleoside analogue), in HIV positive and KSHV- MCD patients, revealed potential results. This procedure generated, among others: control of symptoms, normalization of lytic activity and control of viral load. Moreover, 86% of patients showed positive clinical responses, with an overall survival rate of 86% at 12 months or longer (Uldrick et al. 2011). A large in vitro study using BCBL-1 cells latently infected with KSHV evaluated viral susceptibility using a real-time PCR–based system following treatment with several antiviral agents—acyclovir, foscarnet, ganciclovir, cidofovir, and adefovir, which primarily target viral DNA polymerase and lytic DNA replication (Sergerie and Boivin 2003). Cidofovir was more efficient in viral restriction/inhibition activity (50% - IC50 = 0.43 µM), followed by Ganciclovir (2.61 µM), Adefovir (18.00 µM), Acyclovir (31.00 µM) and Foscarnet (34.15 µM).

In addition to traditional antiviral approaches, recent advances have broadened the therapeutic landscape, as it is important to emphasize that no targeted therapies currently exist for KSHV-associated diseases. A recently published study identified non-cytotoxic inhibitors of KSHV lytic replication using the Medicines for Malaria Venture (MMV) Pandemic Response Box. The compound MMV1645152 was identified as a promising inhibitor of lytic replication, suppressing the expression of KSHV IE, E, and L genes and blocking the production of infectious viral particles at non-cytotoxic concentrations in KSHV-infected cell lines, with or without EBV coinfection (Okpara et al. 2024). These finding highlights that, beyond nucleoside analogs and other classical antiviral agents already evaluated for infection control, new classes of molecules may act through distinct and highly specific mechanisms, offering promising alternatives for future clinical interventions, particularly in scenarios of refractoriness or toxicity associated with conventional antivirals.

The HIV aspartyl protease inhibitor nelfinavir interferes with the assembly and maturation of newly formed KSHV virions, resulting in a significant reduction in extracellular virus production. The proposed mechanism involves activation of the integrated stress response (ISR), leading to decreased synthesis of essential viral proteins such as the capsid protein ORF26 (Li et al. 2024). Notably, inhibition requires only short drug exposure, suggesting potential for unconventional therapeutic strategies (e.g., localized treatment).

In summary, although these findings highlight important advances in the identification of compounds with activity against KSHV, the number of studies truly dedicated to developing effective antivirals remains very limited. Unlike other well-studied herpesviruses, KSHV/HHV-8 still lacks established, virus-specific therapies, reflecting challenges such as viral latency, the diversity of clinical manifestations, and limitations of current experimental models.

### Target therapy

Important specific drugs can be seen in the treatment regimen for KS and other types of disorders developed by KSHV, however, there are no ones that target viral antigens. Molecules expressed in both replicative phases can modulate numerous host cellular pathways, such as the mechanistic target of rapamycin (mTOR). Through the evaluation of the treatment with Everolimus (mTOR inhibitor in the induction of apoptosis of PEL cells) in PEL-NHL cells, it was possible to observe dose-dependent apoptosis and Everolimus triggered down-regulation in the expression of LANA with subsequent inhibition in KSHV lytic reactivation (Mohanty et al. 2017).

This type of therapy is an important way to contain the infection caused by KSHV, as it can target other immunological targets expressed by the virus, such as homologues of chemokines and inflammatory cytokines (Fig. 4). In addition, it is clear that classical chemotherapy strategies are increasingly becoming ineffective, since, among others, they eliminate and/or delay healthy cells and are unable to reach fundamental molecular pathways for KSHV replication and survival.

Recently, a mechanism was described through which KSHV “hijacks” host-cell metabolic pathways to sustain its oncogenesis: the virus activates the de novo nucleotide synthesis pathway via the enzyme CAD, while also promoting glycolytic reprogramming through NF-κB deamidation, thereby favoring cellular proliferation and viral persistence. The dysregulation of host-cell metabolism by KSHV is proposed here, for the first time, as one of the Hallmarks of Cancer (Fig. 5). In the study reported in 2024, the complex formed between vCyc and the cellular kinase CDK6 results in CAD phosphorylation, activating this metabolic axis and promoting tumorigenicity in KSHV-related lymphoma models (Wan et al. 2024). Both genetic and pharmacological inhibition of CDK6 or CAD dramatically reduced lytic viral replication and prevented tumor growth in vitro and in vivo. These findings indicate that the CDK6/CAD pathway represents a therapeutically exploitable “vulnerable point” in KSHV-associated malignancies— such as KS — opening new avenues for targeted therapies that interfere with virus-induced cellular metabolic reprogramming rather than acting directly on viral antigens. In an effort to expand therapeutic options for AEKS and CKS, the NCT04065152 study proposed the use of Talimogene laherparepvec (T-VEC) — the first oncolytic immunotherapy approved by the FDA — administered via intralesional injection into measurable cutaneous lesions (ClinicalTrials.gov 2019). The study design includes 12 treatment cycles over a period of up to six months. The rationale is based on the high immunogenicity of KSHV-associated tumor cells, which aligns with the mechanism of action of T-VEC: direct lysis of injected tumor cells and induction of a systemic antitumor immune response, potentially affecting non-injected lesions as well. Thus, this trial represents an innovative strategy that combines oncolytic virotherapy and immunotherapy, with the potential to fill a therapeutic gap in KS management, particularly in patients whose underlying immunosuppression cannot be reversed.

### Gene editing and regulation

LANA is the most extensively studied latency-associated antigen of KSHV, as it is consistently expressed in all latently infected cells during lifelong infection. In recent years, the gene-editing technique CRISPR-Cas9 has gained prominence in studies of KSHV infection. Targeted editing of the LANA gene using CRISPR-Cas9 resulted in a reduction of KSHV latency, as evidenced by a decline in viral DNA levels following gene editing (Tso et al. 2019; Haddad et al. 2021). A study described an innovative gene therapy strategy based on the selective expression of suicide genes in KSHV-infected cells. This approach exploited a LANA-dependent regulatory element to drive herpes simplex virus thymidine kinase (HSV-tk) expression specifically in KSHV-positive cells using plasmid or lentiviral constructs. HSV-tk phosphorylates the prodrug ganciclovir, generating a toxic triphosphate metabolite that induces cell death in proliferating cells. In vitro assays in latently infected cell lines, including BCBL-1 and BC-3, showed that activation of the LANA promoter resulted in robust and selective HSV-tk expression, leading to cell death upon ganciclovir treatment without cytotoxic effects in KSHV-negative cells (Inagaki et al. 2025). Consistently, in murine xenograft models of primary effusion lymphoma, this therapeutic strategy significantly reduced tumor burden and improved survival following ganciclovir administration. These findings demonstrate that regulatory elements derived from KSHV latency genes can serve as highly specific platforms for targeted gene therapy in virus-associated malignancies such as KS and PEL.As shown in Fig.4, the protein homolog vFLIP expressed by KSHV during the latent phase, and also becomes a candidate, among others, for being a potent activator of the NF-kB pathway and inhibitor of apoptosis. In studies of its gene regulation using the phosphorylation inhibitor of this pathway, Bay 11–7082, resulted in the induction of apoptosis in lymphoma cells (Sadek et al. 2020), which also places vFLIP in the ranking of KSHV molecules with remarkable capacities to be therapeutic targets.

Thus, we can conclude that treatments for patients with KS are the same as decades ago, with classic treatments being the most used, although many efforts have been made with ongoing clinical trials. On the other hand, it can be seen that there are not enough therapeutic studies aimed at inhibiting/inactivating KSHV, that is, eliminating the KS-inducing agent and other disorders. Thus, tumor progression during or after the classic therapeutic regimen, as well as the worst prognosis, including the observation of septic shock and consequent death, continues to be a reality.

### Emergence of new technological approaches for the treatment of viral agents

Infectious diseases are one of the leading causes of mortality worldwide, posing a significant global impact on health and socioeconomic development. Throughout the 21 st century, numerous viral epidemic events have been recorded, including the COVID-19 pandemic in 2020, which highlighted the vulnerability of global health systems and the need for rapid and effective responses to infectious agents. Beyond acute infections, oncogenic viruses also represent a major public health challenge, as they often establish persistent infections that can lead to cancer development. It is estimated that approximately 13% of all cancers worldwide are caused by viral infections, including KSHV (de Martel 2020), making it a significant concern. KSHV is associated with other DLPs, such as PEL and MCD, further expanding its clinical impact. However, the COVID-19 pandemic has introduced new challenges for patients with KSHV-associated diseases, as SARS-CoV-2 infection can exacerbate immunosuppression and increase the risk of KSHV reactivation, leading to more severe clinical manifestations of KS and other complications (Shiny Talukder et al. 2024; Reactivation 2024). Moreover, the pandemic has underscored the importance of specific and broad-spectrum antiviral therapies, which can be adapted to combat oncogenic viruses like KSHV. Therefore, the development of targeted therapies for KSHV is critically necessary, as viral inactivation could not only reduce viral load and tumor progression but also mitigate severe clinical manifestations in KS patients, especially in a global scenario where co-infections, such as COVID-19, can complicate clinical management. The integration of innovative approaches, such as the use of therapeutic aptamers and gene-editing-based therapies, may offer new perspectives for controlling KSHV and its associated pathologies, representing a crucial advancement in the fight against virus-related cancers.

Promising studies focused on the search for new technologies to inactivate many types of virus have been carried out, such as: (i) the interaction of nanotechnology with viral agents, offering promising tools in diagnosis and therapy, associated with drug delivery (Singh 2017); (ii) AstraZeneca–Oxford vaccine based on adenovirus and mRNA-based vaccines (Pfizer–BioNTech and Moderna) approved for emergency use in the COVID-19 pandemic (Baden 2021; Polack 2020); (iii) cold plasma as an efficient solution for virus inactivation has been applied to viruses such as norovirus, adenovirus, hepatitis A virus and HIV (Filipić et al. 2020); (iv) multiepitopes-based vaccine projection directed to the main glycoproteins of KSHV, through immunoinformatics approach (Chauhan et al. 2019); (v) Creation of two experimental KSHV vaccines—an mRNA vaccine with K8.1, and another based on ferritin nanoparticles with K8.1 fragments. Both induced an immune response (antibodies + T cells) and reduced virus replication/reactivation (Yang et al. 2024).

### Advances in the development of therapies for KSHV inactivation

**Fig. 8:**
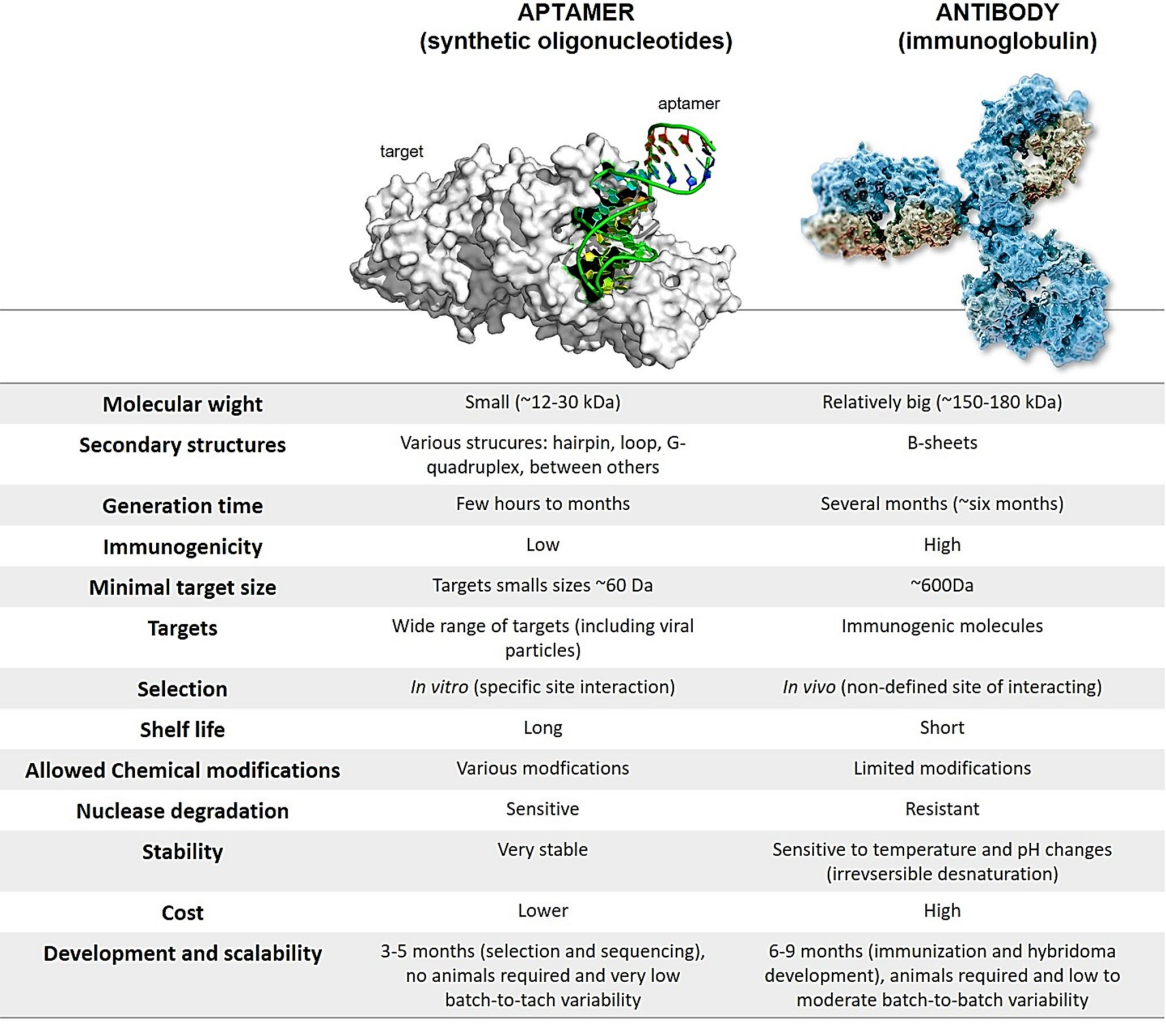
Comparison of the properties of aptamers and antibodies. Adapted from: Hali M.A., Elsherbiny M.E. et al., 2019 and The Scientist.

The field of synthetic biology was highlighted in 2003, and its definition is due to the incorporation of different research areas including engineering, physics, and computer science, with the purpose of building new biological components (Fig. 8). In this context, aptamers are single-stranded synthetic oligonucleotides of DNA, RNA, or XNA (xenonucleic acids) with 40 to 80 nucleotides, that bind with high specificity to a molecular target and are therefore strong candidates for diagnostic tests and therapy. As an advantage, aptamers could be structurally modified, in order to improve them in terms of biological stabilization, favoring its pharmacokinetics, pharmacodynamics, and safety for preclinical studies (Fig. 8). The most common strategies in the chemical modifications of aptamers include (i) modifications on the terminals of nucleic acids, (ii) modifications on the phosphodiester linkage, (iii) modifications on the sugar ring and on the bases, (iv) modifications on the 3`end capping with inverted thymidine and (v) PEGylation. Some aptamers have undergone structural changes and are now in pre-clinical studies: OPN-R3 (target: osteopontin), AS1411 (target: nucleoline), ESTA (target: E-selectin), ARC1779 (target: blood clotting protein - VWF), Pegaptanib (target: VEGF), among others. These aptamers showed safety with no toxicity, no adverse effects and good tolerance in the trials (Kovacevic et al. 2018).

**Fig. 7:**
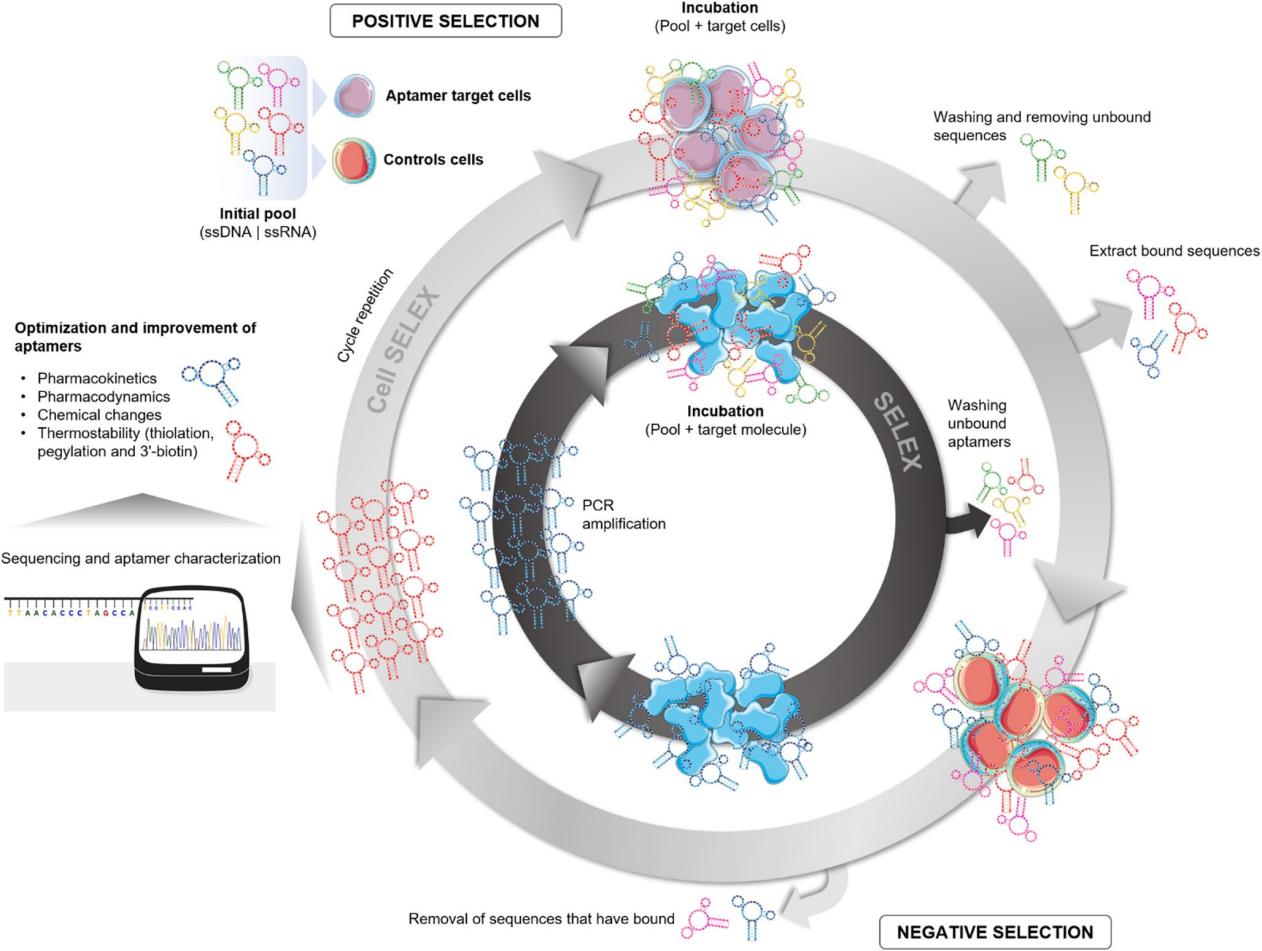
Cell Selex x SELEX. Aptamers are single-stranded DNA or RNA oligonucleotides selected for high affinity and specificity toward target molecules, including proteins or cells. The illustration depicts the SELEX process, including both conventional SELEX for protein targets and cell-SELEX for whole-cell targets, involving iterative cycles of incubation with the target, removal of unbound sequences, elution of bound aptamers, and amplification to enrich target-specific sequences. Some elements of the illustration were created using Servier Medical Art

Aptamers are selected through an in vitro process called “Systematic Evolution of Ligands through EXponential enrichment” (SELEX) which can result in the recognition of a wide variety of target molecules, from small structures to macromolecules. Besides the classic SELEX, cell-based SELEX (Cell-SELEX) could also be applied as a method of selection of aptamers in whole cells. The use of Cell-SELEX has advantages, as it is based on the selection of aptamers against molecules of living cells that maintain their native conformations and, in this way, preserve their biological functions. It is important to highlight that at the end of the aptamer selection rounds during SELEX procedure, the selected pool of specific aptamers could be identified by sequencing methods, and then, be used as individual aptamers, being then suitable for optimization, as discussed above (Allemailem 2020; Chai et al. 2011) (Fig.7).

Despite recent advances in drug repurposing and broad-spectrum antiviral strategies, approaches directed toward KSHV-specific molecular targets remain scarce. The few studies involving aptamers illustrate this scenario and, although they suggest translational potential, they present important limitations.

The first example is the anti-nucleolin aptamer AS1411, investigated in an NIH-funded project aimed at evaluating its therapeutic potential. AS1411 recognizes nucleolin (a cellular protein involved in proliferation and transcriptional regulation) and forms G-quadruplex structures, and has been explored in models related to KS/KSHV (Sharma-Walia 2024). However, because it targets a host protein rather than viral components, its effects on KSHV are indirect, limiting its applicability as a virus-specific antiviral.

The second case involves an aptamer directed against HIV TAR RNA, which reduced the progression of KSHV infection mediated by exosomes derived from HIV-positive cells (Chen et al. 2020). This effect, however, results from interference with HIV-dependent coinfection mechanisms, without direct interaction with the KSHV replication cycle or its molecular targets.

### Targeting virus by aptamer technology

Virus-derived and host-associated targets represent promising candidates for aptamer-based therapeutic and diagnostic strategies. In the context of KSHV infection and KS, aptamers offer an attractive yet largely unexplored platform, particularly given the absence of virus-specific targeted therapies and the complex interplay between viral latency, immune evasion, and oncogenic signaling. Herpes simplex virus (HSV) glycoprotein D (gD) plays a notable role in the interaction with cell receptors and during virus entry into the host cell. Two RNA aptamers (apRNA) were shown to bind with high specificity to HSV-1 gD, preventing gD-receptor interaction, thus inhibiting viral entry into HVEM cells. Unlike gD, there are other herpesvirus surface glycoproteins that are shared by virus family members, including KSHV, and one of them is gB. DNA aptamers against gB were developed and tested, presenting binding stability and good physical-chemical properties. Cytomegalovirus encodes a regulatory protein (pUL84) that, among others, plays a key role in initiating virus lytic replication. An aptamer targeting pUL84 exhibited intracellular activity, with decreased cytomegalovirus replication in primary fibroblast (Kaiser 2009). Based on these advances, several KSHV proteins emerge as rational targets for aptamer development, including envelope glycoproteins involved in viral entry (such as gB, gH/gL, and K8.1), as well as latent and lytic regulatory proteins essential for episomal maintenance and reactivation, including LANA and RTA. Targeting such molecules could enable interference with viral entry, persistence, or reactivation—key processes underlying KS pathogenesis.

Patients in the KS-HIV group present a dual viral infection, in which one promotes immunosuppressive infection, and the other develops KS. Therefore, it should be highlighted the relevance of studies with THEAPTAs also targeting the HIV virus. HIV-1 protease enzyme plays a role in viral morphogenesis, and its inhibitors are potentially affective, but are followed by multiple severe adverse effects and resistance. The development of anti-HIV-1 protease apRNA for use in gene therapy have been reported, with specificity and inhibiting capacity of viral replication (Duclair et al. 2015). Other THEAPTAs studies with potential application for HIV virus proteins include: (i) the gp120 glycoprotein of the viral envelope, with specific apRNAs that enable the blocking of entry into the cell, via binding/neutralization of gp120 (Mufhandu et al. 2012; Dey et al. 2005); (ii) the apRNA that targets the gag structural polyprotein showed inhibition of extracellular capsid183; (iii) the Rev protein, a post transcriptional protein, which is an aptamer target with antiviral action via delivery of cationic liposome (Konopka et al. 1998); (iv) nucleocapsid protein (NC), that is involved in the viral encapsulation process, was suppressed after binding to aptamers (Kim and Jeong 2004). Some HIV constituent enzymes also become targets of THEAPTAs due to their great importance in infection, aptamers selected specifically for these enzymes play a restrictive role in late events of viral replication, inhibiting the action of RT, integrase and protease (Duclair et al. 2015; Michalowski et al. 2008; Ojwang et al. 1995). Although these studies were conducted in other viral systems, they provide strong proof-of-concept that aptamers can effectively target conserved herpesviral glycoproteins and regulatory proteins, supporting their potential applicability to KSHV.

Recent advances in synthetic biology have strengthened the use of aptamers as highly specific molecular tools capable of modulating essential viral processes. Current strategies include aptamer–siRNA conjugates for transcriptional regulation and aptamers engineered to recognize critical viral proteins—such as Rav, nucleocapsid (NC) proteins, reverse transcriptase (RT), integrases, and proteases of HIV (Bala et al. 2018), as well as gB of HCMV (Kumar 2021)and gD of HSV (Gopinath 2012).

Recent studies have demonstrated substantial progress in the diagnostic field. Lateral-flow devices based exclusively on aptamer–aptamer recognition, without the use of antibodies, have achieved up to 100% sensitivity and over 90% specificity in clinical samples (Dilek Çam et al. 2024). In parallel, recent reviews have highlighted the integration of aptamers into fluorescence-based sensors, electrochemical detection systems, and SPR platforms, showing superior stability, precision, and robustness for detecting respiratory viruses across diverse biological matrices (Feng et al. 2024).

In the therapeutic domain, studies published this year indicate that aptamers can directly block proteins essential for viral entry or replication, and that their conjugation with nanomaterials—giving rise to aptamer-guided nanotherapies—enhances stability, targeted delivery, and antiviral potency (Zhang et al. 2025). Additionally, advanced chemical modifications (2’-F, 2’-O-methyl, LNA) have generated aptamers with high affinity, biosensing capabilities, and targeted drug-delivery potential, combined with low immunogenicity and strong nuclease resistance. These features consolidate aptamers as “synthetic antibodies” with extensive structural modularity (Borkar et al. 2025).

Despite their promising features, aptamer-based strategies face important limitations in the context of KSHV. These include the lack of experimentally validated KSHV-specific aptamers, challenges related to in vivo stability and delivery, potential off-target effects, and the absence of suitable preclinical models for KS. Addressing these limitations will be critical for translating aptamer technologies into clinically relevant applications for KSHV-associated diseases.

Collectively, the evidence positions aptamers as highly promising platforms for developing diagnostic and therapeutic strategies directed against KSHV. Considering the lack of specific and effective antivirals and the urgent need for selective, low-toxicity approaches capable of modulating critical viral and cellular events, aptamers emerge as versatile tools with real potential to innovate the diagnosis and clinical management of KS and other KSHV-associated diseases. Importantly, our recent search on ClinicalTrials.gov identified only 115 interventional studies related to KS—94 involving HIV positive or HIV negative patients and only 10 addressing forms such as CKS/AEKS/HIV^−^—with 74 studies already completed. Moreover, only 8 of the 94 HIV negative related studies were actively recruiting phase II trials, underscoring the limited attention given to the development of specific or personalized therapies for the multiple forms of KS.

Furthermore, the expanding body of aptamer research across diverse biomedical fields underscores their versatility, tunable structural properties, and favorable physicochemical characteristics (Fig. 8). These features position aptamers as promising candidates for antiviral strategies, including blockade of viral envelope glycoproteins, modulation of intracellular regulatory targets, and targeted drug-delivery approaches.

Notably, despite advances in synthetic molecules and targeted therapies against related herpesviruses, no aptamers have yet been specifically developed against KSHV. This gap highlights an unmet translational need and emphasizes the importance of experimental studies investigating aptamer-based strategies targeting the etiological agent of KS.

## Conclusions

KS is a hybrid and complex cancer, and even with the knowledge of its pathogenic agent and its possible pathways demonstrated here, many hypotheses raised have not yet been legitimized in vivo, in silico and/or *in vitro*. However, even after almost 3 decades of identification of KS and KSHV, the development of efficient therapies is still a challenge for the scientific and medical community, especially in endemic regions and in immunocompromised populations. Despite considerable advances in the understanding of KSHV biology and the oncogenic mechanisms it triggers, the translation of this knowledge into targeted therapies is still limited. It is well known, and explored here, that KSHV can promote a variety of clinical pathologies by regulating the control of the cellular machinery and the individual’s immune system, mainly through the expression of its oncoproteins. Therefore, we analyzed the Clinical Trials database for therapeutic studies on cancer, and particularly for KSHV. Therefore, some deficiencies can be concluded:

I. Although there are mainly studies with HIV-related patients, there are no clinical studies for the AKS group and only 10, with the CKS, AEKS or HIV groups;
II. 64% of the clinical studies analyzed indicate that they have already been concluded, that is, they ended normally and the participants are no longer being examined or treated (that is, the last visit of the last participant occurred);
III. Regarding the period of initiation of the studies, in the years 2012 to 2022 the majority of clinical studies covered interventions based on immunotherapy, while in the period 2001 to 2011 the dominant methods were CCTP;
IV. A group of unconventional therapies (considered innovative for the treatment of KS) was observed as an interventional basis for 14 studies involving topical treatments, radiopharmaceuticals, cardiac glycoside, local compression therapy, ONCOVI, photodynamic therapy, among others;

The emergence of new technologies, such as CRISPR-Cas9 gene editing and the use of therapeutic aptamers, offers promising prospects for the future. These approaches have the potential to revolutionize the treatment of KS, not only by inhibiting viral replication, but also by modulating the cellular pathways that support tumor progression. The integration of these technologies with personalized precision medicine strategies may lead to more effective and less toxic treatments, significantly improving the quality of life of patients.

This study significantly advances our understanding of the oncogenic mechanisms of KSHV, offering critical insights that pave the way for the development of novel diagnostic tools and targeted antiviral therapies. By elucidating key pathways and molecular interactions, our findings provide a robust foundation for future research aimed at early detection and effective management of KSHV-associated malignancies, ultimately improving patient outcomes and quality of life. With the continuous development of cutting-edge technologies and an integrated approach, we can envision a horizon where KS, and other oncovirus-associated diseases, will no longer be a death sentence, but a manageable condition, with treatments that not only prolong life, but also preserve its quality.

## Future perspectives

KS remains a major clinical challenge, particularly in immunocompromised populations and in regions where KSHV is endemic. Although significant progress has been made in elucidating the viral oncogenic machinery, the translation of these discoveries into effective antiviral therapies is still limited. Our review demonstrates that KSHV manipulates multiple cancer hallmarks through its latent and lytic oncoproteins, highlighting a set of viral determinants—such as LANA, vFLIP, vCyclin, vGPCR, and glycoproteins involved in viral entry—that remain largely unexplored as therapeutic targets.

In this context, future directions should prioritize the development of strategies that directly target the virus, rather than relying exclusively on broad-spectrum antivirals or nonspecific immunotherapies. Aptamer-based technologies represent an especially promising frontier: beyond their diagnostic applications, rationally designed aptamers could inhibit viral entry, block latency maintenance, or disrupt signaling pathways essential for KSHV-driven tumorigenesis. Gene-editing tools, such as CRISPR-Cas9, also emerge as powerful candidates capable of selectively disrupting viral genomes in latently infected cells.

Integrating these next-generation platforms with precision-medicine approaches and high-resolution omics technologies may enable the identification of actionable vulnerabilities in KSHV-associated malignancies. Importantly, the scarcity of clinical trials specifically focused on KSHV underscores the urgency of investing in translational research that bridges molecular discoveries and therapeutic development.

Therefore, the future outlook is optimistic: it is expected that, in the short to medium term, clinical studies will bring concrete advances, directing efforts toward the elimination of KSHV and, consequently, the prevention of KS and other severe disorders associated with this oncogenic virus.

## Data Availability

The datasets generated and/or analyzed during this study are available in the World Clinical Trials Database repository, [https://clinicaltrials.gov/](https:/clinicaltrials.gov).
